# CRYAB promotes osteogenic differentiation of human bone marrow stem cells via stabilizing β‐catenin and promoting the Wnt signalling

**DOI:** 10.1111/cpr.12709

**Published:** 2019-10-22

**Authors:** Bin Zhu, Feng Xue, Guangyi Li, Changqing Zhang

**Affiliations:** ^1^ Department of Orthopaedics Shanghai Jiao Tong University Affiliated Sixth People's Hospital Shanghai China

**Keywords:** bone, CRYAB, osteogenic differentiation, Wnt/β‐catenin signaling

## Abstract

**Objectives:**

The osteogenesis differentiation of human bone marrow stem cells (BMSCs) is essential for bone formation and bone homeostasis. In this study, we aim to elucidate novel molecular targets for bone metabolism diseases.

**Materials and methods:**

The dataset GSE80614 which includes mRNA expression profile during BMSCs osteogenic differentiation was obtained from the GEO database (https://www.ncbi.nlm.nih.gov/geo/). The osteogenic differentiation of BMSCs was measured by ALP staining, AR staining and expression of osteogenic markers in vitro. For in vivo assay, we seeded BMSCs onto beta‐tricalcium phosphate (β‐TCP) and transplanted them into muscle pockets of nude mice. Luciferase assay, co‐immunoprecipitation assay and in vitro ubiquitination assay were carried out to investigate the molecular mechanism.

**Results:**

We found that α‐B‐crystallin (CRYAB) expression was elevated during the process of BMSCs osteogenic differentiation. Further studies showed that upregulation of CRYAB significantly enhanced the osteogenic differentiation, while downregulation of CRYAB suppressed it. CRYAB regulated BMSCs osteogenic differentiation mainly through the canonical Wnt/β‐catenin signalling. In addition, we found that CRYAB could physically interact with β‐catenin and protect it from ubiquitination and degradation, which stabilized β‐catenin and promoted the Wnt signalling.

**Conclusions:**

The present study provides evidences that CRYAB is an important regulator of BMSCs osteogenic differentiation by protecting β‐catenin from ubiquitination and degradation and promoting the Wnt signalling. It may serve as a potential therapeutic target for diseases related to bone metabolism.

## 1 INTRODUCTION

Human bone marrow stem cells (BMSCs) exhibit great capacity to differentiate into numerous mature cell types under the guidance of several factors including microenvironment, molecular and genetic mediators.^1^, ^2^, ^3^ Osteoblasts, as one of the products of BMSCs differentiation, play a key role in bone formation and bone homeostasis.^4^, ^5^, ^6^ The dysfunction of BMSCs osteogenic differentiation will lead to bone metabolism‐related diseases.^7^, ^8^ Thus, it is crucial to elucidate the underlying mechanism of BMSCs osteogenic differentiation.

Canonical Wnt signalling has been revealed to be critical in maintaining homeostasis and embryonic development in human.^9^, ^10^, ^11^ The transduction of Wnts signals through the plasma membrane relies on the frizzled protein, which is a member of G protein‐coupled receptors family.^12^, ^13^ When the Wnt ligands are absent, a multiprotein complex is composed by β‐catenin, AXIN, adenomatous polyposis coli (APC), glycogen synthase kinase 3β and casein kinase 1. In the presence of Wnt ligands, the protein complex is phosphorylated and decomposed, and β‐catenin is released. Then, the released β‐catenin is transferred into the nucleus and combines with T‐cell factor/lymphoid enhancer factor (TCF/LEF) to start the following transcriptional activities.^14^, ^15^ Increasing studies have demonstrated that the canonical Wnt/β‐catenin pathway is vital to osteogenic differentiation of BMSCs and bone metabolism.^16^, ^17^, ^18^

The ubiquitin‐proteasome system is essential for the elimination of several short‐lived proteins. Commonly, targeted proteins are labelled by ubiquitin molecules through a sequence of enzymatic reactions, including E1 (ubiquitin‐activating enzymes), E2 (ubiquitin‐conjugation enzymes) and E3 (ubiquitin ligases), and are eventually degraded into peptides by proteasomes. Evidences have verified that β‐catenin, a key factor of Wnt/β‐catenin signalling pathway, is also a target for the ubiquitin‐proteasome system, and that the degradation of β‐catenin directed by ubiquitin‐proteasome system plays important roles in tumour suppression,^19^, ^20^, ^21^ bone marrow stem cell osteogenic differentiation,^22^, ^23^ et al

α‐B‐crystallin (CRYAB) has been reported as a major structural protein of the eye lens and is abundant in kidney, central nervous system, skeletal muscle and heart.^24^, ^25^ As a member of the small heat‐shock protein (sHSP) family, CRYAB plays important roles in cellular development, differentiation and apoptosis inhibition.^26^, ^27^, ^28^ In addition, CRYAB also acts as a molecular chaperone and functions in the recognition, binding and refolding of several unfolded proteins, and protects them from degradation.^29^, ^30^ In this study, we discovered that the CRYAB expression is gradually increased in BMSCs during the osteogenic differentiation process. Further experiments revealed that CRYAB exhibits a promotion effect on BMSCs osteogenic differentiation both in vitro and in vivo. In addition, we demonstrated that CRYAB could regulate the Wnt/β‐catenin signalling by protecting β‐catenin from ubiquitination and degradation. In summary, these findings indicate that CRYAB acts as a regulator of BMSCs osteogenic differentiation and may serve as an underlying therapeutic target for diseases related to bone homeostasis.

## 2 MATERIALS AND METHODS

### 2.1 Microarray analysis

The mRNA expression profile during osteogenic differentiation process of human bone marrow stem cells was included in the dataset GSE80614. We obtained the dataset from the GEO database (https://www.ncbi.nlm.nih.gov/geo/) and analysed the differentially expressed mRNAs at different time points (3 and 4 days vs 1 and 2 hours). |logFC| > 2 and *P*‐value < .01 was set as threshold.

### 2.2 Cell culture

Human bone marrow stem cells (BMSCs) were got from a 43‐year‐old donor who suffered an severe trauma and underwent amputation and were maintained in α‐modified essential medium (α‐MEM; Sigma‐Aldrich) that contained 10% foetal bovine serum (FBS) (Gibco) and 100 µg/mL penicillin‐streptomycin sulphate (Sigma‐Aldrich) at 37°C in a 5% CO_2_ humidified incubator.

### 2.3 In vitro differentiation assays

For the in vitro adipogenic and osteogenic differentiation assays, BMSCs were treated with adipogenic differentiation medium (Cyagen) or osteogenic differentiation medium (Cyagen) follow the operating instruction in 24‐well plates for 7 or 14 days. The differentiation medium was refreshed every 2 days. Oil Red O staining, Western blot and qRT‐PCR were used to measure the adipogenic differentiation efficacy. Alizarin red (AR) staining, alkaline phosphatase (ALP) staining, Western blotting and qRT‐PCR were used to measure the osteogenic differentiation efficacy.

### 2.4 RNA isolation and qRT‐PCR assays

The isolation of total RNA from BMSCs was performed using TRIzol reagents (Invitrogen) and isolated the total RNA was then quantified using Nanodrop 2000 (Thermo). The PrimerScriptRT Reagent (TaKaRa) was used according to the operating instruction for reverse‐transcription of cDNA. The reactions of real‐time PCR were performed with the aid of SYBR Green kit (TaKaRa) using ABI HT7900 (Applied Biostralia). The gene expression levels were normalized to β‐actin. Primer sequences are listed in Table S1.

### 2.5 Western blot analysis and Co‐immunoprecipitation

Cells were lysed in Cell Lysis Buffer (Sigma‐Aldrich) on ice and denatured by heating at 95°C for 15 minutes with the addition of loading buffer (BioTNT). The samples were separated using a 10% SDS‐PAGE gel (EpiAyme) by electrophoresis at 120 V for 75 minutes. Then, the proteins were transferred through electroblotting at 280 mA for 70 minutes to a PVDF membrane (Millipore). After blocking in 5% non‐fat milk for 60 minutes, the membrane was incubated with primary antibodies at 4°C for 12 hours, followed by incubation with the secondary antibody at room temperature for 1 hour. Image Quant LAS 4000 (GE Healthcare) was used to confirm the expression levels of proteins. The expression of β‐actin was used as control.

An immunoprecipitation kit (Abcam) was used for co‐immunoprecipitation assay according to the operating instruction. Briefly, lysis buffer was pre‐cooled on ice and the Protease Inhibitor Cocktail was added for the preparation of cell lysis. The supernatant of the cell lysis was collected after centrifuged at 1000 g for 15 minutes, and the primary antibodies were added and incubated at 4°C for one night. Then, the Protein A/G Sepharose beads were added and incubated at 4°C for 1 hour and then separated by centrifugation at low speed. The beads were washed for three times and the precipitated proteins were eluted followed by Western blotting analysis.

The antibodies are listed in Table S2.

### 2.6 Plasmid transfection and reporter gene activity assay

For siRNA transfection, Lipofectamine 2000 (Invitrogen, California, USA) was used according to the manufacturer's instructions and the cells were collected to assess the efficacy of transfection by qRT‐PCR or Western blotting 24 hours later. For virus productions, 293T cells were transfected with 3.6 µg envelope plasmid, 9 µg packaging plasmid and 12 µg targeting plasmids using Lipofectamine 2000, and the viruses were harvested and filtered 48 hours later. The infection of BMSCs was performed with the addition of polybrene follow the operating instruction. For TOPFlash reporter assay, 293T cells were transfected with β‐galactosidase, CRYAB‐specific siRNA or corresponding control siRNA and Super (8×) TOPFlash plasmid using Lipofectamine 2000. Serum starvation was performed for overnight 24 hours after the transfection, and the osteogenic differentiation medium was then used for cell culture. The cells were lysed 48 hours later and a luciferase assay system (Promega) was used to measure the luciferase activity.

### 2.7 In vitro ubiquitination assay

Cells were lysed using cell lysis buffer after treated with a proteasome inhibitor, MG 132, for 8 hours, and the lysis was incubated with antibodies for 3 hours at 4°C. Then the Sepharose beads of protein A/G were added to the lysis and rotated gently for 10 hours at 4°C. The beads were collected and washed three times with centrifugation at low speed. The SDS‐loading buffer was used to elute the immunoprecipitated proteins at 95°C for 3 hours, and Western blotting assay was applied for analysis.

### 2.8 Animal studies

Eight‐week‐old male nude mice were employed for animal studies in this experiment, and all of these mice were kept in a specific‐pathogen‐free condition. Beta‐tricalcium phosphate (β‐TCP; Bio‐lu, 5 × 5 × 3 mm) with BMSCs (5 × 10^6^) seeded on was transplanted and buried into muscle pockets on the posterior limb of the mice. Forty‐two mice were divided into seven groups, namely sh‐Control group, sh‐CRYAB group, Vector group, CRYAB group, sh‐Control + Vector group, sh‐CRYAB + Vectro group and sh‐CRYAB + β‐catenin group. Corresponding kind of BMSCs was used in each group. The mice were put to death 8 weeks later, and the transplants were collected from the posterior limbs for histological analysis. 10% EDTA was used to decalcify the transplants after fixation in 10% formalin for 3 days. The samples were then sliced using Leica RM2235 for H&E (Haematoxylin and Eosin) staining. BIOQUANT OSTEO software (BIOQUANT) was used to collect the images. All experiments were subject to approval by the Ethics Committee of Shanghai Sixth People's Hospital.

### 2.9 Statistical analysis

All results were replicated independently for three times. SPSS 16.0 (IBM Corporation) was used to complete the statistical analysis. Two‐tailed Student's *t* test was used to evaluate the statistical differences between two groups, and one‐way analysis of variance (ANOVA) was used to evaluate the statistical differences among more than two groups. Values of *P* < .05 were considered statistically significant. All values are presented as means ± SD (standard deviation).

## 3 RESULTS

### 3.1 CRYAB expression is upregulated during the process of BMSCs osteogenic differentiation

**Figure 1:**
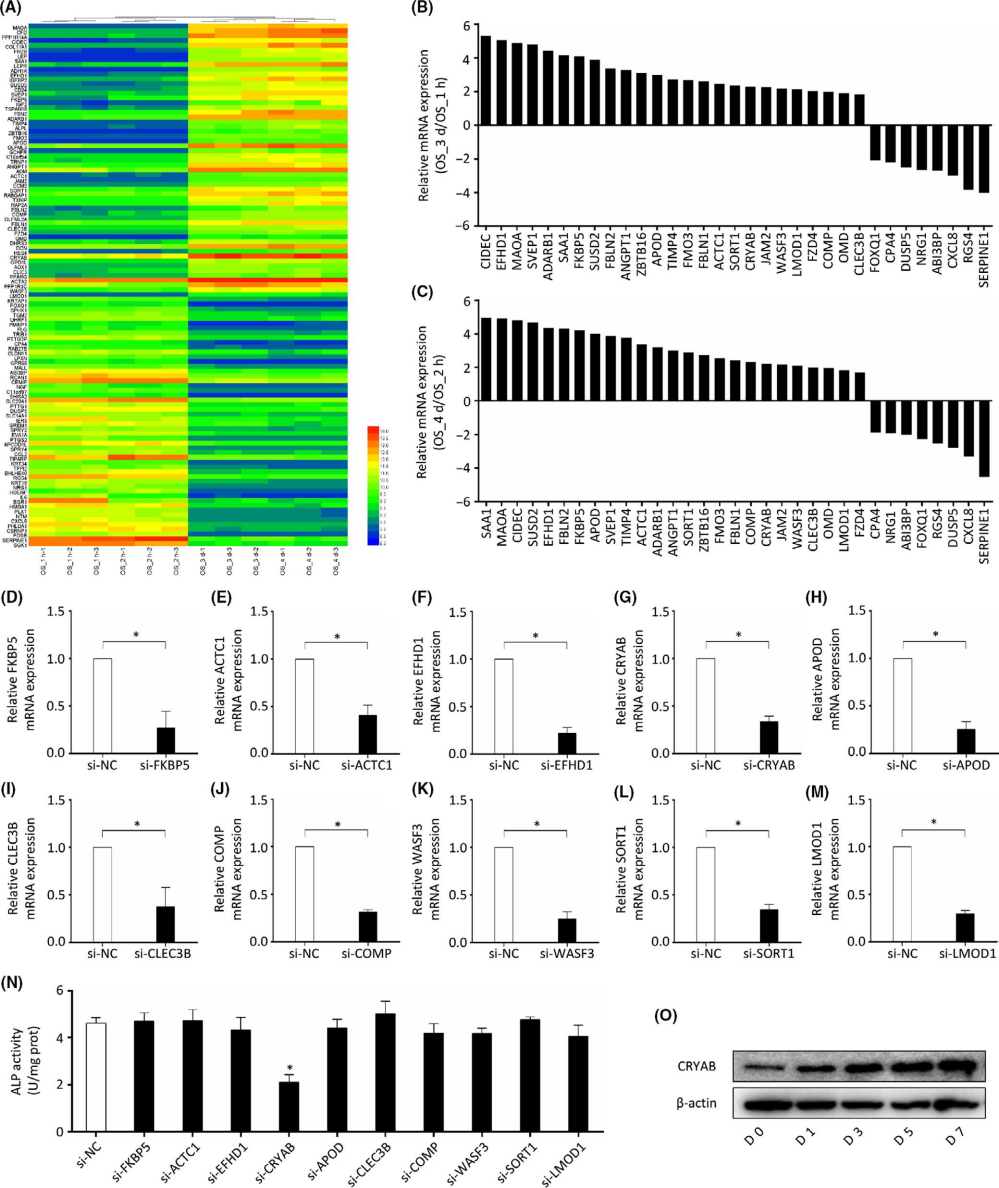
CRYAB expression is upregulated during the process of BMSCs osteogenic differentiation. A, A total of 109 differentially expressed genes (52 downregulated genes and 57 upregulated genes) were identified when comparing the differentiation time of 3 or 4 d to that of 1 or 2 h. B&C, Eight downregulated genes and twenty‐five upregulated genes were chosen to further validate the dataset using qRT‐PCR. D‐M, Ten upregulated genes were chosen for functional screen in BMSCs and their expression were downregulated using specific siRNA. N, ALP activity was measured after BMSCs were treated with specific siRNA. O, The expression of CRYAB in BMSCs was measured using Western blotting during the process of osteogenic differentiation

In the present study, the dataset GSE80614 was analysed to explore the differentially expressed genes at different time points during BMSCs osteogenic differentiation process. A total of 109 differentially expressed genes (including 52 downregulated genes and 57 upregulated genes) were identified when comparing the differentiation time of 3 or 4 days to that of 1 or 2 hours (Figure 1A). The bioinformatics analysis of these differentially expressed genes is shown in Figure S1. Then, eight downregulated genes and 25 upregulated genes were chosen to further validate the dataset. As determined by qRT‐PCR analysis, the expression of these genes was consistent with the dataset (Figure 1B and C). Previous studies have reported several of the above‐mentioned genes (CIDEC, MAOA, SAA1, SVEP1, FBLN2, ADARB1, ZBTB16, ANGPT1, FBLN1, FZD4, OMD, ABI3BP, RGS4, CXCL8) are closely related to bone homeostasis and the process of osteogenic differentiation,^31^, ^32^, ^33^, ^34^, ^35^, ^36^, ^37^, ^38^, ^39^, ^40^, ^41^, ^42^, ^43^, ^44^ and these studies further supported the reliability of the dataset. To identify the BMSCs osteogenic differentiation‐related genes, we chose 10 of the other upregulated genes, namely EFHD1, FKBP5, APOD, ACTC1, SORT1, COMP, CLEC3B, CRYAB, WASF3, LMOD1, and downregulated their expression by specific siRNA in BMSCs (Figure 1D‐M). The results showed that the downregulation of CRYAB expression significantly impaired the capacity of BMSCs osteogenic differentiation as determined by ALP activity (Figure 1N). Furthermore, we also detected the CRYAB protein expression level and found that it was gradually increased along with the course of osteogenic differentiation (Figure 1O). Therefore, we speculated that CRYAB might play a critical role in human BMSCs osteogenic differentiation process.

### 3.2 The role of CRYAB in osteogenic differentiation in vitro

**Figure 2:**
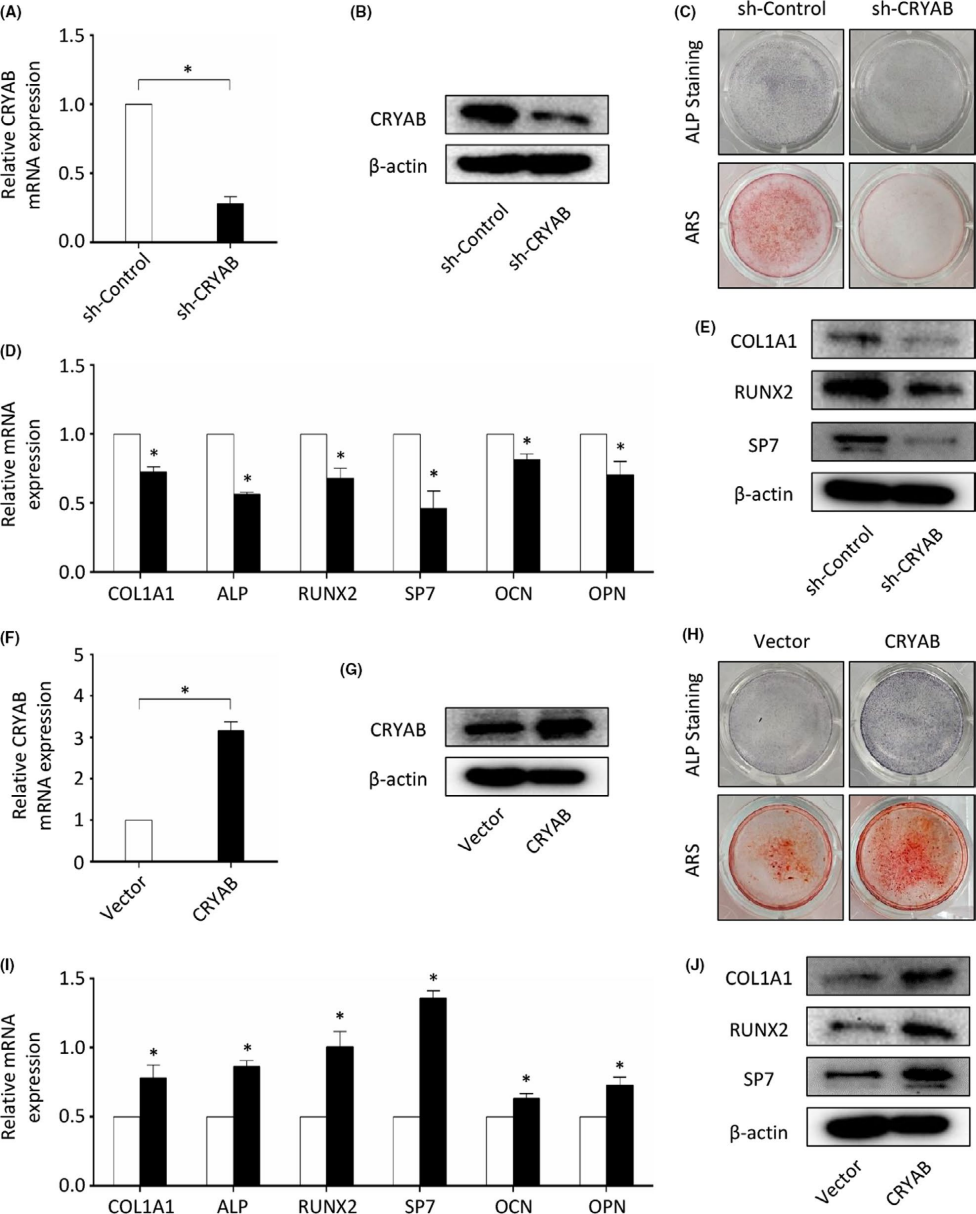
The role of CRYAB in osteogenic differentiation in vitro. A&B, qRT‐PCR and Western blotting were used to determine the efficacy of CRYAB‐specific shRNA. C, The osteogenic capacity of CRYAB‐downregulated BMSCs was measured by alizarin red staining and ALP staining at day 14 or day 7 after treated with osteogenic induction media. D, The expression of osteogenic markers at mRNA level was measured by qRT‐PCR. E, The expression of osteogenic markers at protein level was measured by Western blotting. F&G, qRT‐PCR and Western blotting were used to determine the efficacy of CRYAB‐specific lentivirus. H, The osteogenic capacity of CRYAB‐upregulated BMSCs was measured by alizarin red staining and ALP staining at day 14 or day 7 after treated with osteogenic induction media. I, The expression of osteogenic markers at mRNA level was measured by qRT‐PCR. J, The expression of osteogenic markers at protein level was measured by Western blotting

To verify our speculation about the role of CRYAB in the process of human BMSCs osteogenic differentiation, we knocked down its expression using a specific shRNA and evaluated the change of osteogenic differentiation capacity using ALP staining at day 7 and alizarin red staining at day 14 (Figure 2A and B). We found that knocking‐down of CRYAB expression significantly inhibited the capacity of BMSCs osteogenic differentiation as evaluated by ALP and AR staining (Figure 2C). We also found that the mRNA and protein expression levels of osteogenic markers, including COL1A1, ALP, RUNX2, SP7, OCN and OPN, were inhibited by knocking‐down of CRYAB expression (Figure 2D and E). Additionally, we also upregulated the CRYAB expression in human BMSCs using specific lentivirus in order to further confirm its role in osteogenic differentiation. Western blotting and qRT‐PCR were used to assess the efficacy of lentivirus (Figure 2F and G), and ALP staining and AR staining were used to assess the capacity of osteogenic differentiation. We found that upregulation of CRYAB expression significantly enhanced the capacity of BMSCs osteogenic differentiation as evaluated by ALP and AR staining (Figure 2H). Additionally, the mRNA and protein expression levels of osteogenic markers, including COL1A1, ALP, RUNX2, SP7, OCN and OPN, were increased by upregulation of CRYAB expression (Figure 2I and J).

### 3.3 The function of CRYAB in osteogenic differentiation in vivo

**Figure 3:**
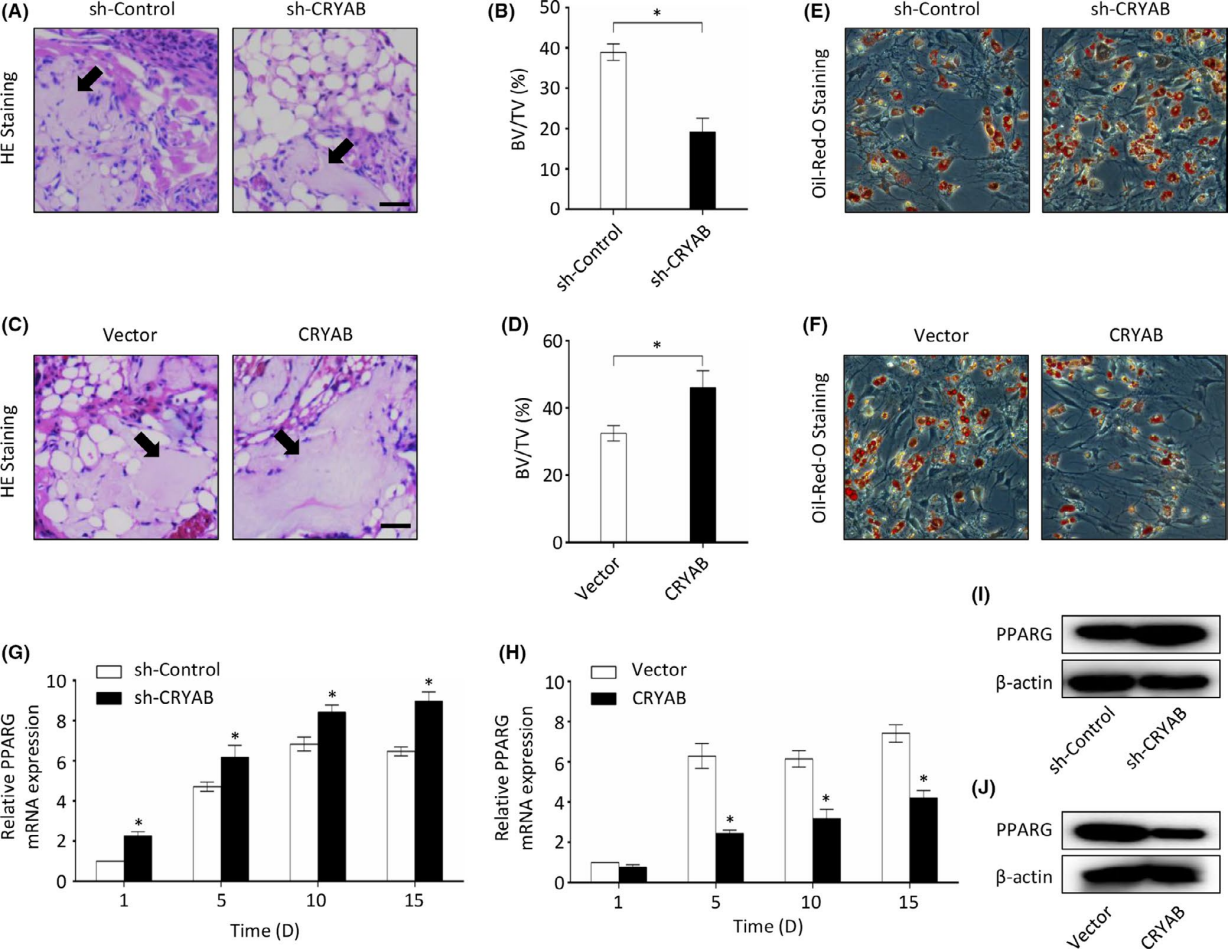
The function of CRYAB in osteogenic differentiation in vivo. A, Representative images of H&E staining of transplants in the sh‐CRYAB group and sh‐Control group. The new formed bone was denoted by black arrows. Scale bar represents 100 µm. B, The bone formation was measured qualitatively. C, Representative images of H&E staining of transplants in the CRYAB group and Vector group. The new formed bone was denoted by black arrows. Scale bar represents 100 µm. D, The bone formation was measured qualitatively. E&F, Representative images of Oil Red O staining in different groups after BMSCs were treated for 14 d with adipogenic induction medium. G&H, The qRT‐PCR was used to measure the expression of PPARG in BMSCs at mRNA level during the adipogenic differentiation process. I&J, The Western blotting was used to measure the expression of PPARG in BMSCs at protein level at day 15 of the adipogenic differentiation

In vivo experiments were performed to further confirm our in vitro findings. BMSCs treated with CRYAB‐specific shRNA or lentivirus to stably down‐ or upregulate its expression were loaded on shaped β‐TCP (tricalcium phosphate), followed by transplantation into muscle pockets on the posterior limb of nude mice. The mice were put to death 8 weeks later, and the transplants were collected from the posterior limbs for histological analysis. We found that less bone tissue was formed in the CRYAB‐downregulated group when compared with the control group (Figure 3A and B), while more bone tissue was formed in the CRYAB‐upregulated group when compared with its control group (Figure 3C and D). Furthermore, we could also find that downregulation of CRYAB expression increased the formation of adipose tissue while upregulation of CRYAB expression decreased the formation of adipose tissue in H&E staining (Figure 3A and C). Therefore, we hypothesized that CRYAB might also play a critical role in human BMSCs adipogenic differentiation.

To further verify our hypothesis, we assessed the adipogenic differentiation efficacy of BMSCs using Oil Red O staining and qRT‐PCR assays after the cells were treated with adipogenic induction medium. It was demonstrated that the adipogenesis of BMSCs was enhanced by downregulating CRYAB expression (Figure 3E), while reduced by upregulating CRYAB expression (Figure 3F). And the downregulation of CRYAB expression significantly enhanced the expression of adipogenic markers, PPARG, CEBPA and ADIPOQ, while the upregulation of CRYAB expression reduced them (Figure 3G‐J, Figure S2).

### 3.4 CRYAB regulates the BMSCs osteogenic differentiation via the WNT signalling

**Figure 4:**
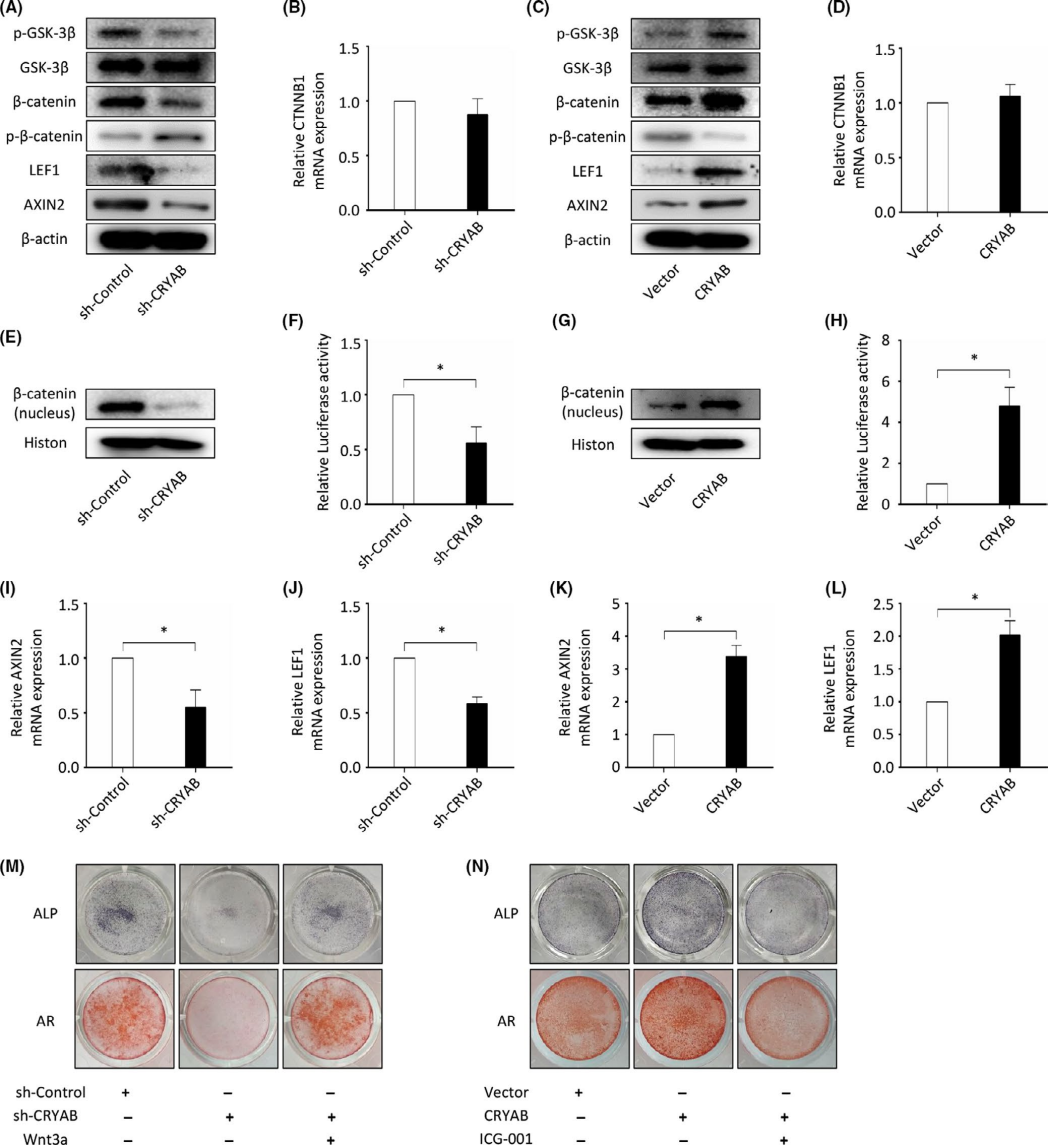
CRYAB regulates the BMSCs osteogenic differentiation via the WNT signalling. A, The expression of GSK‐3β, p‐GSK‐3β (Ser9), β‐catenin, p‐β‐catenin (Ser33/37/Thr41), LEF1 and AXIN2 at protein level in sh‐CRYAB group and sh‐Control group was measured by Western blotting. B, The expression of CTNNB1 at mRNA level in sh‐CRYAB group and sh‐Control group was measured by qRT‐PCR. C, The expression of GSK‐3β, p‐GSK‐3β (Ser9), β‐catenin, p‐β‐catenin (Ser33/37/Thr41), LEF1 and AXIN2 at protein level in CRYAB group and Vector group was measured by Western blotting. D, The expression of CTNNB1 at mRNA level in CRYAB group and Vector group was measured by qRT‐PCR. E, The expression of nuclear β‐catenin at protein level in sh‐CRYAB group and sh‐Control group was measured by Western blotting. F, Relative TOPFlash luciferase activity was measured in sh‐CRYAB group and sh‐Control group. G, The expression of nuclear β‐catenin at protein level in CRYAB group and Vector group was measured by Western blotting. H, Relative TOPFlash luciferase activity was measured in CRYAB group and Vector group. I‐L, The expression of AXIN2 and LEF1 at mRNA level was measured by qRT‐PCR. M, AR staining and ALP staining showed that the restricted osteogenic capacity of BMSCs resulting from the downregulation of CRYAB expression was rescued by the treatment with Wnt3a. N, AR staining and ALP staining showed that the enhanced osteogenic capacity of BMSCs resulting from the upregulation of CRYAB expression was reduced by the treatment with ICG‐001

The canonical Wnt signalling has been proven to be vital for human bone homeostasis and human BMSCs osteogenic differentiation. To explore whether CRYAB influenced the process of osteogenesis through the Wnt signalling, we firstly measured the expression of β‐catenin, a core element of the Wnt/β‐catenin pathway. We found that the downregulation of CRYAB expression significantly decreased β‐catenin protein level as measured by Western blotting, and upregulation of CRYAB expression increased it (Figure 4A and C). However, the alteration of CRYAB expression hardly influenced the β‐catenin mRNA level as measured by qRT‐PCR (Figure 4B and D). In addition, the phosphorylation level of β‐catenin was increased by downregulation of CRYAB, and decreased by upregulation of CRYAB (Figure 4A and C). Besides, we found that the upregulation of CRYAB promoted the nuclear accumulation of β‐catenin, and the downregulation of CRYAB suppressed it (Figure 4E and G). Then, we measured the GSK‐3β phosphorylation level at Ser9, which is a vital step for the activation of canonical Wnt signalling.^45^, ^46^ We found that the expression level of GSK‐3β phosphorylated at Ser9 was reduced by downregulation of CRYAB expression and elevated by upregulation of CRYAB expression, while the total GSK‐3β levels almost kept the same (Figure 4A and C). In addition, we employed the TOPFlash reporter assay to further assess the effect of CRYAB on the Wnt signalling in 293T cell line. As we have shown in Figure 4F and H, the luciferase activity was significantly increased by upregulation of CRYAB and decreased by downregulation of CRYAB. To further confirm the effect of CRYAB on the Wnt signalling, we determined the regulation effect of CRYAB on the Wnt signalling target genes, including AXIN2 and LEF1. As measured by qRT‐PCR, the mRNA expression of these target genes was upregulated by the upregulation of CRYAB expression, and downregulated by the downregulation of CRYAB expression (Figure 4I‐L). The results were also confirmed in protein level by Western blotting (Figure 4A and C). Besides, as determined by ALP and AR staining, the enhanced osteogenic capacity of BMSCs resulting from the upregulation of CRYAB expression was reduced by the treatment with ICG‐001, an inhibitor of the Wnt signalling; and the restricted osteogenic capacity of BMSCs resulting from the downregulation of CRYAB expression was rescued by the treatment with Wnt3a, an activator of the Wnt signalling (Figure 4M and N).

### 3.5 CRYAB is essential for β‐catenin stabilization

**Figure 5:**
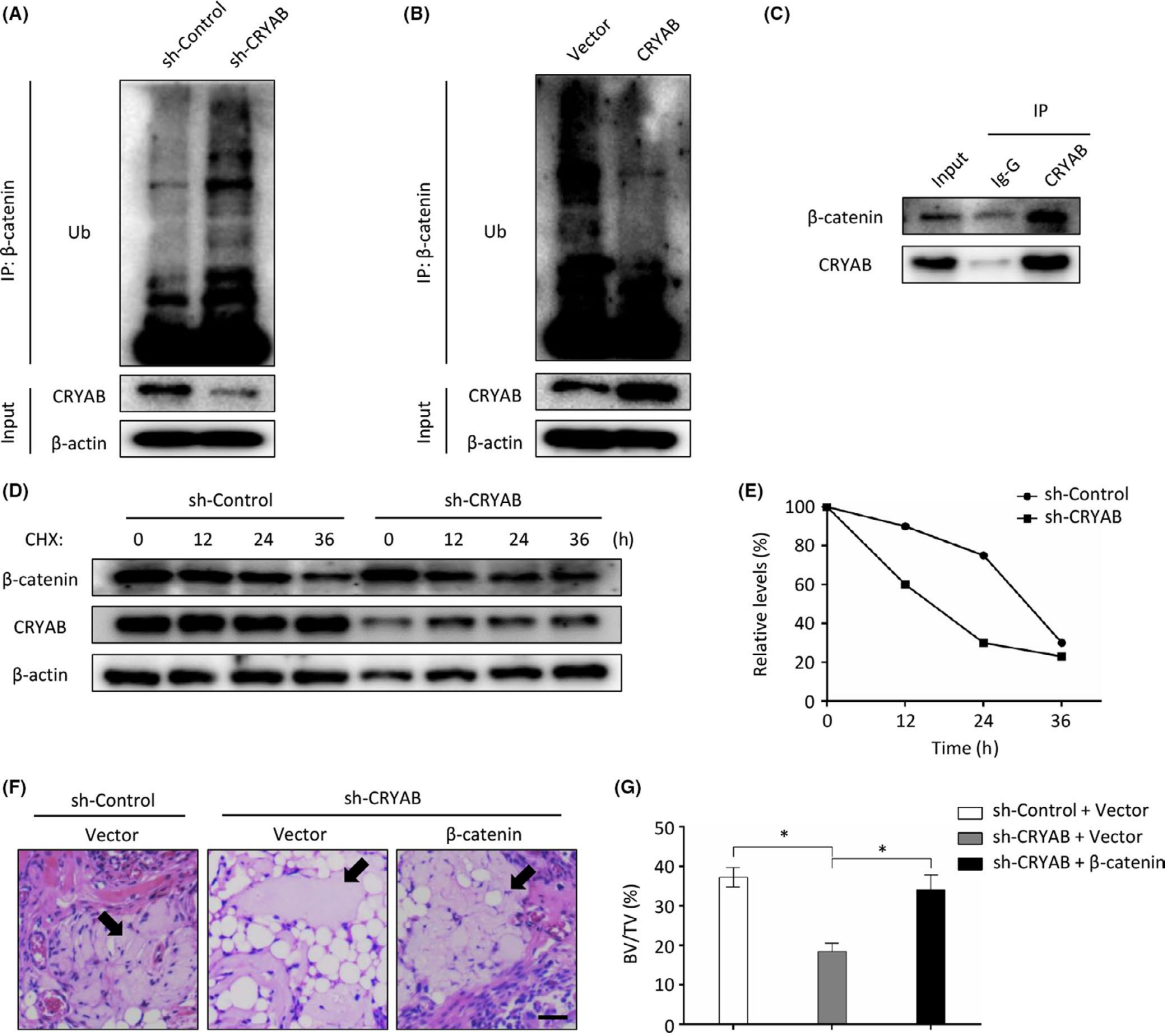
CRYAB is essential for β‐catenin stabilization. A&B, The ubiquitination level of β‐catenin was enhanced by the downregulation of CRYAB and reduced by the upregulation of CRYAB. C, CRYAB could physically interact with β‐catenin in human BMSCs as determined by co‐immunoprecipitation. D&E, The stability of β‐catenin was determined after BMSCs were treated with a protein synthesis inhibitor, cycloheximide (CHX), for indicated hours. F, Representative images of H&E staining of transplants from different groups. The new formed bone was denoted by black arrows. Scale bar represents 100 µm. G, The bone formation was measured qualitatively

The ubiquitin‐proteasome system has been verified to play essential roles in regulating the stability, subcellular localization and activity of proteins. Since the alteration of CRYAB expression significantly influenced the protein level of β‐catenin without changing its mRNA level (Figure 4A‐D), we speculated that the degradation of β‐catenin by the ubiquitin‐proteasome system may be influenced by CRYAB. To verify our speculation, the co‐immunoprecipitation assay was carried out and we found that CRYAB could physically interact with β‐catenin in human BMSCs (Figure 5C). The ubiquitination of β‐catenin was examined in BMSCs after treatment with a proteasome inhibitor, MG132, for 8 hours. We found that the ubiquitination level of β‐catenin was enhanced by the downregulation of CRYAB and reduced by the upregulation of CRYAB (Figure 5A and B). In addition, the stability of β‐catenin was determined after BMSCs were treated with a protein synthesis inhibitor, cycloheximide (CHX), for indicated hours. We found that the downregulation of CRYAB significantly reduced the half‐life of β‐catenin (Figure 5D and E).

Besides, CRYAB‐downregulated BMSCs were transfected with β‐catenin‐lentivirus or the corresponding control lentivirus, and the cells were loaded on β‐TCP and transplanted into muscle pockets on the posterior limb of nude mice. Eight weeks later, the mice were put to death and the transplants were harvested for histological analysis. As shown in Figure 5F and G, the overexpression of β‐catenin could rebuild the osteogenic differentiation capacity of CRYAB‐downregulated BMSCs.

**Figure 6:**
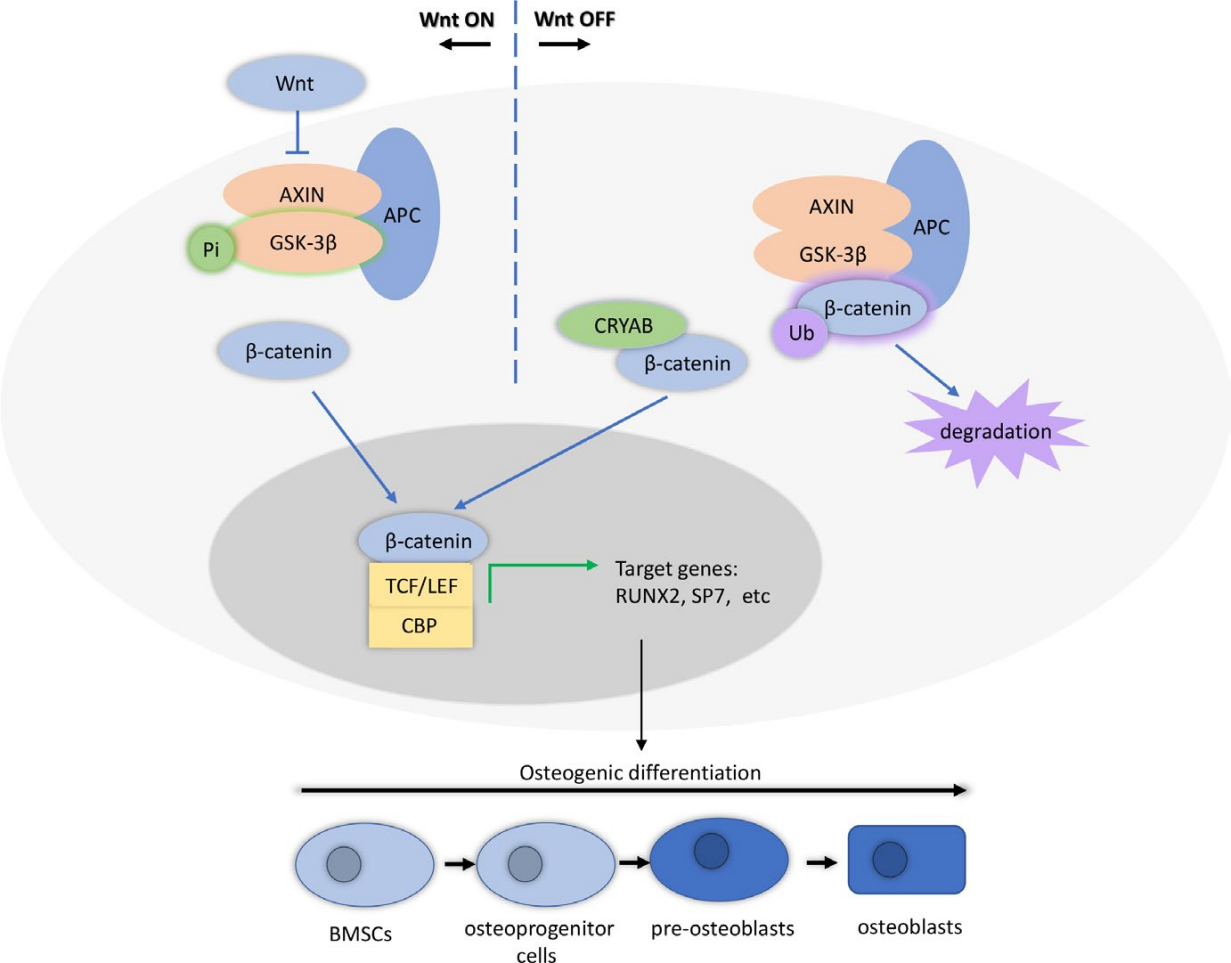
CRYAB promotes osteogenic differentiation of human bone marrow stem cells via stabilizing β‐catenin and promoting the Wnt signalling

Additionally, the regulatory network of CRYAB on the osteogenic differentiation of BMSCs is shown in Figure 6.

## 4 DISCUSSION

Bone is a metabolically active organ of human and is continuously remodelled via the formation of new bone by osteoblasts and the resorption of old bone by osteoclasts.^47^, ^48^ The dysfunction of osteoblasts, which are the products of BMSCs osteogenic differentiation, may induce a decline in bone formation and an imbalance of bone homeostasis, and it may eventually cause bone metabolism‐related diseases.^49^, ^50^ Thus, it is necessary to figure out the molecular mechanism involved in BMSCs osteogenic differentiation, which may provide us potential targets for the improvement of therapeutic approaches for bone metabolism‐related diseases.

Total mRNA expression of human BMSCs cultured in osteogenic differentiation medium was measured at different time points by van de Peppel J. and his colleagues, and the mRNA expression profile was included in the dataset GSE80614.^51^ We studied this dataset in the current research by comparing the expression profiles of BMSCs (3 & 4 days vs 1 & 2 hours) and identified 52 downregulated and 57 upregulated genes. Twenty‐five upregulated and eight downregulated genes were selected to examine the reliability of the microarray. The reliability of the dataset was further verified given that several of the selected genes have been assessed before and were proven to be strongly linked to osteogenic differentiation of BMSCs and bone homeostasis.^31^, ^32^, ^33^, ^34^, ^35^, ^36^, ^37^, ^38^, ^39^, ^40^, ^41^, ^42^, ^43^, ^44^ Then, functional screen was performed by ALP activity assays after target genes were knocked down by specific siRNA. We found that the ALP activity was significantly reduced by the downregulation of CRYAB expression in BMSCs. These findings revealed that CRYAB plays a role in the osteogenic differentiation of BMSCs.

As a member of the small heat‐shock protein family, CRYAB possesses several important roles. In cardiomyocytes and vascular endothelial cells, CRYAB has been shown to exhibit a protective role against apoptosis.^52^, ^53^, ^54^, ^55^ The expression of CRYAB has also been proven to be closely related to neurological diseases, including Alexander's disease, Parkinson's disease and Alzheimer's disease.^56^, ^57^, ^58^ In addition, CRYAB is deemed as an oncogenic gene for its protection effect on cancer cells from chemotherapeutic drugs.^54^, ^59^ Previous studies on mass spectrum and oligonucleotide microarrays also found an alteration of CRYAB expression level during osteogenic differentiation of human BMSCs, which is consistent with our findings from the dataset http://www.ncbi.nlm.nih.gov/geo/query/acc.cgi?acc=GSE80614.^60^, ^61^ However, the exact role of CRYAB on osteogenic differentiation of BMSCs has seldomly been studied. To further explore the role of CRYAB on osteogenic differentiation of BMSCs, we performed in vitro and in vivo studies. In the in vitro study, we discovered that the expression level of CRYAB in BMSCs was closely related with their capacity of osteogenesis as measured by alizarin red staining and ALP staining. In vivo experiments using a BMSCs‐transplantation assay in nude mice further confirmed the observation. Furthermore, we found an inverse relationship between the expression of CRYAB and the capability of BMSCs adipogenic differentiation. These findings manifest that CRYAB is essential for BMSCs osteogenic differentiation.

The canonical Wnt/β‐catenin signalling has been verified to exhibit a vital effect on BMSCs osteogenic differentiation and bone metabolism.^62^, ^63^ Kuci S and his colleagues noticed that the expression of CRYAB and the activity of Wnt signalling were both increased during the osteogenic differentiation process of BMSCs.^64^ In this study, we found that the Wnt/β‐catenin signalling was also crucial for the CRYAB‐regulated osteogenic differentiation of BMSCs. Since β‐catenin is a core component of the Wnt/β‐catenin signalling and acts a core function of this pathway,^65^ we further explored the relationship of CRYAB and β‐catenin. We found that CRYAB could physically interact with β‐catenin and protect it from ubiquitination and degradation.

In conclusion, we found that the expression of CRYAB is upregulated in the osteogenic differentiation process of BMSCs. CRYAB acts as a regulator of BMSCs osteogenic differentiation, and the regulatory effect is achieved by stabilizing β‐catenin and regulating the Wnt signalling. These findings illustrate that CRYAB might be a new regulator of BMSCs osteogenic differentiation and may serve as an underlying therapeutic target for diseases related to bone homeostasis.

## CONFLICTS OF INTEREST

The authors declare no conflicts of interest.

## Data Availability

The data that support the findings of this study are openly available in GEO database at https://www.ncbi.nlm.nih.gov/geo/query/acc.cgi?acc=GSE80614.^66^
