# Supplementary material for: CRYAB promotes osteogenic differentiation of human bone marrow stem cells via stabilizing β‐catenin and promoting the Wnt signalling
Source: Cell Prolif. 2019 Oct 22;53(1):e12709. doi: 10.1111/cpr.12709 (PMC6985673; doi:10.1111/cpr.12709)

**CRYAB Promotes Osteogenic Differentiation of Human Bone Marrow Stem Cells via Stabilizing β-catenin and Promoting the Wnt Signaling**

Bin Zhu, Feng Xue, Guangyi Li, Changqing Zhang

Department of Orthopaedics, Shanghai Jiao Tong University Affiliated Sixth People’s Hospital, Shanghai, China.

Table 1. The sequences of the primers

| Gene Symbol | Forward primer | Reverse primer | Product length |
| --- | --- | --- | --- |
| MAOA | CCCGGAGTATCAGCAAAA | CAAAGGGTGGGAAGGAC | 127 |
| CIDEC | GAGCCAGGGGATGAGAAA | GGAGAGGGACTTGGGGTAG | 100 |
| SAA1 | CTATGATGCTGCCAAAAGG | CTCCTGCCCCATTCATT | 141 |
| SVEP1 | TGGAACATGGACATCACCT | CCCTCTGGACAGGAACAA | 102 |
| EFHD1 | GATGCCCCGAGAAGAGA | CTTGGACACTGGGAGGAA | 125 |
| FKBP5 | GGGTAGGGGAAGGAGGATA | GGAACTGGGGAGACATGAG | 119 |
| FBLN2 | TTCTGGACCCCTCCTCTG | CATCACCCAGCCTCACC | 132 |
| SUSD2 | TACTACGGCACCGCCAA | AGGGCATTCCTGTCTCCTC | 115 |
| ANGPT1 | TCTCTGCCTGTAAGTGTCCA | ACTGGTATTGCTACCTTGCC | 142 |
| ZBTB16 | TTCCTCTCGCCAAAGACC | CTCCAGCATCTTCAGGCA | 150 |
| APOD | GAAGTGCCCCAATCCTC | GCAGCGTCCATTCTCAA | 106 |
| ADARB1 | GGCCATTCATTCAGGTTTT | TCACCCACATCCATTCG | 128 |
| TIMP4 | CTCCCAAACCCCATTAGTC | CGCCATTTCTCCCCTAC | 150 |
| FMO3 | TTATCCTTGGGACATGCTG | GCATTCATCTGCTTCACG | 102 |
| JAM2 | AAATGCCTAATGGCTGGA | CTTGGGTTTGTGTTTGACC | 107 |
| FBLN1 | GACCACGGGCATTTGAG | TGAACAGCAGCATTCCATC | 113 |
| ACTC1 | AGATGATTGGCTGGGGTAT | TGGGTGCTTACCTCTTCTG | 136 |
| SORT1 | TCTCATGGCCTTCGTCA | GGTTGGCCTGGAAACTAAA | 116 |
| WASF3 | GACCTCCTCGCTGCTATTC | GCTGTACTCCACGGCAA | 135 |
| CRYAB | GAGTCCCTTCTACCTTCGG | CCATGCACCTCAATCACA | 174 |
| LMOD1 | GTTGTCGGGTCTTATTGGC | TCCTTGGCCCTTCTGTG | 117 |
| FZD4 | CCAGAACCTCGGCTACAA | GGATGAGCGGTGTGAAAG | 101 |
| COMP | CAGGGAAGGGAGATCGTG | CGCATAGTCGTCATCCGT | 126 |
| OMD | GGGAAAAGAAAGAAGTAAGCC | GAAAACACTCGGGTTGAATTA | 120 |
| CLEC3B | GGCAAGACCGAGAACTGC | GGCTACACGATCCCGAAC | 110 |
| FOXQ1 | CGGAAGAGGACTCCGGAAAAG | GTCGTACCCTCTTCCTTCGC | 144 |
| CPA4 | TGGACGGCAAGGAAGAT | CAGCACCAATGCAGGAG | 184 |
| DUSP5 | GGACCAGGAGAAAAGGCA | CCCGAAGCAACAGAGACA | 109 |
| NRG1 | TGAATGGAGGGGAGTGCT | CAGGCAGAGACAGAAAGGG | 150 |
| ABI3BP | CCTGAAGCACCTGAATCC | TTAGTTTTGGGACGTGGAC | 131 |
| CXCL8 | GACATACTCCAAACCTTTCCA | AACTTCTCCACAACCCTCTG | 162 |
| RGS4 | CTTGCCTGCTCATGCTC | ATGCGTGTGTTTCACTGG | 108 |
| SERPINE1 | TAGGAGCAGAAATGCAAGG | CAGCCGGAAATGACACA | 111 |
| COL1A1 | CCTGCTGGCAAGAGTGGT | GCCCTGTTCGCCTGTCT | 135 |
| ALP | CTCGCCACTGTCCTGCT | AGTCCGGGTTCTCCTCCT | 125 |
| RUNX2 | GCGGTGCAAACTTTCTCCAG | TGCTTGCAGCCTTAAATGACTC | 114 |
| SP7 | CCCCATCTCCCTTGACTG | TTCCCCAAAGAGCACATCT | 136 |
| OCN | CACACTCCTCGCCCTATTG | TACCTCGCTGCCCTCCT | 124 |
| OPN | CCGTGGGAAGGACAGTT | TTGCTCTCATCATTGGCTT | 108 |
| PPARG | CAGGCCGAGAAGGAGAA | CTTTGGTCAGCGGGAAG | 133 |
| CEBPA | AGCACGATCAGTCCATCCC | TTAGCAGAGACGCGCACA | 143 |
| ADIPOQ | CTTCTGTTCTGCCTTCCG | GGCTTGGGGATACGAGA | 119 |
| CTNNB1 | TGCTAAATGACGAGGACCA | ATCTGAGGAGAACGCATGA | 106 |
| AXIN2 | GCAGCTTCCGTGAGGAT | ACAGGCATGGGTTTGGT | 109 |
| LEF1 | CCCACACAACTGGCATC | TTGGCTCCTGCTCCTTT | 126 |

Table 2. Antibodies

| Proteins | Cat.No. | Company |
| --- | --- | --- |
| CRYAB | ab13497 | Abcam |
| Collagen I | ab34710 | Abcam |
| RUNX2 | #12556 | Cell signaling technology |
| Sp7 | ab22552 | Abcam |
| Ubiquitin | ab7780 | Abcam |
| β-catenin | ab32572 | Abcam |
| p-β-catenin (S33/S37/T41) | #9561 | Cell signaling technology |
| GSK-3β | ab32391 | Abcam |
| p-GSK-3β (Ser9) | ab75814 | Abcam |
| LEF1 | ab137872 | Abcam |
| Axin2 | #5863 | Cell signaling technology |
| PPARG | ab45036 | Abcam |
| Histon | #4499 | Cell signaling technology |
| β-actin | HRP-60008 | Proteintech |
| Secondary antibody | 111-035-003 | Jackson |

**FIGURE S1**. The bioinformatics analysis of the 109 differentially expression genes. A, GO Molecular Function analysis of the differentially expressed genes. B, GO Biological Process analysis of the differentially expressed genes. C, GO Cellular Component analysis of the differentially expressed genes. D, KEGG pathway analysis of the differentially expressed genes.


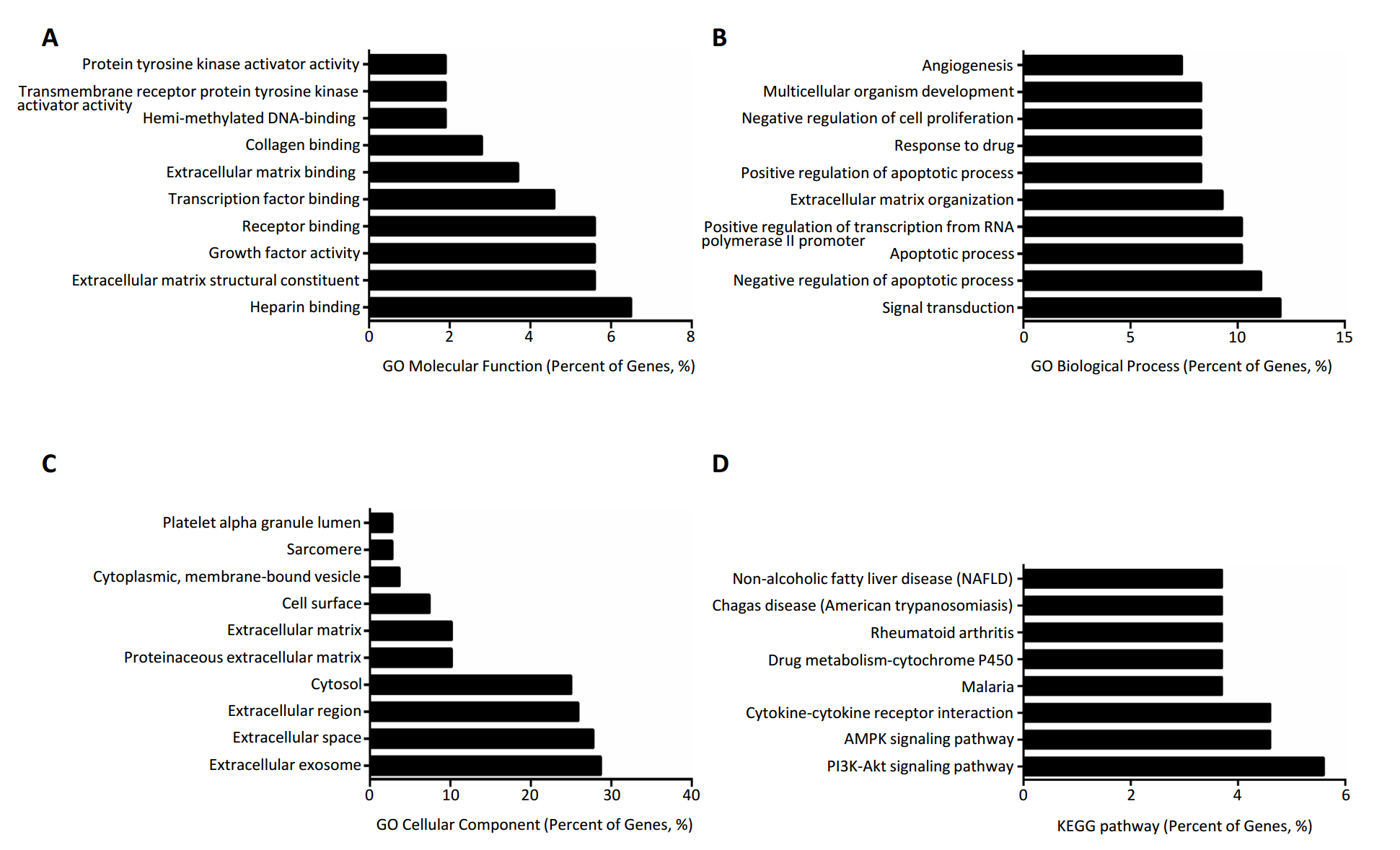


**FIGURE S2**. The qRT-PCR was used to measure the expression of CEBPA and ADIPOQ in BMSCs at mRNA level during the adipogenic differentiation process.


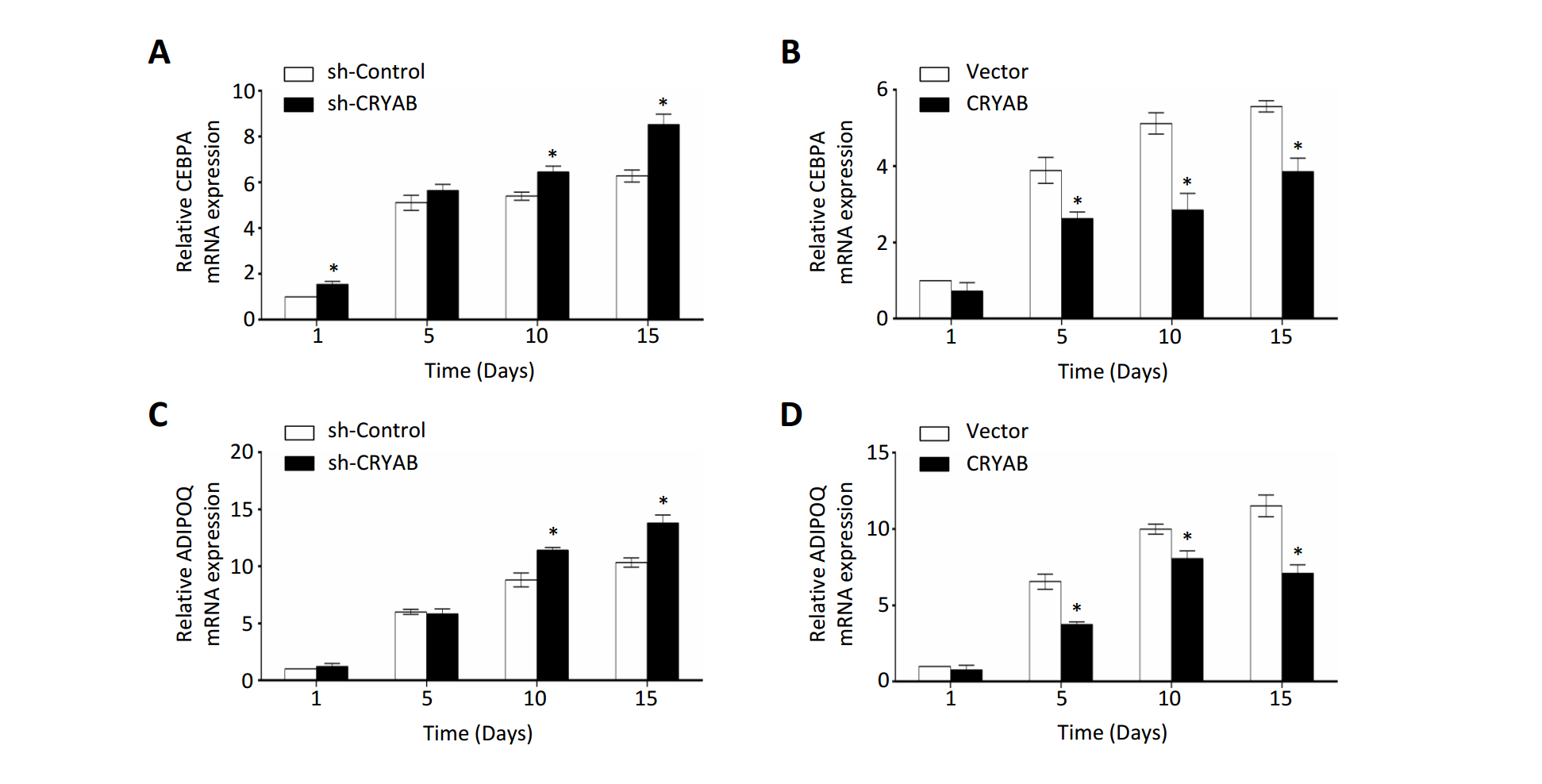

Supplement: Supplementary file 1 [file CPR-53-e12709-s001.docx]
